# Adherence to the Mediterranean Diet Is a Strong Predictor of Glycemic and Lipidemic Control in Adults with Type 2 Diabetes: An Observational Study from a Tertiary Hospital in Greece

**DOI:** 10.3390/nu18020285

**Published:** 2026-01-16

**Authors:** Aristeidis Vavitis, Ioanna A. Anastasiou, Dimitris Kounatidis, Eleni Rebelos, Nikolaos Tentolouris

**Affiliations:** 1 Department of Nutrition and Dietetics, Harokopio University of Athens, 17671 Athens, Greece; 2 First Department of Propaedeutic and Internal Medicine, Laiko General Hospital, Medical School, National and Kapodistrian University of Athens, 11527 Athens, Greece; anastasiouiwanna@gmail.com (I.A.A.); dimitriskounatidis82@outlook.com (D.K.); eleni.rebelos@utu.fi (E.R.); ntentol@med.uoa.gr (N.T.); 3 Department of Clinical and Experimental Medicine, University of Pisa, 56126 Pisa, Italy

**Keywords:** glycemic control, lipidemic control, Mediterranean diet adherence, Mediterranean Diet Score, type 2 diabetes

## Abstract

**Background/Objectives**: Type 2 diabetes (T2D) is a chronic metabolic disorder closely linked to cardiovascular disease and obesity and notably influenced by lifestyle and dietary patterns. The Mediterranean diet has well-established benefits across multiple cardiometabolic risk factors, including those relevant to diabetes. This study aimed to investigate the degree to which adults with T2D adhere to a Mediterranean dietary pattern and to examine how such adherence relates to glycemic and lipidemic regulation. **Methods:** This cross-sectional study included 100 adults with T2D (54 men and 46 women). Adherence to the Mediterranean diet was assessed using the Mediterranean Diet Score (MDS). Demographic, anthropometric, lifestyle, and clinical data were collected, and glycemic and lipid parameters were analyzed. Associations between Mediterranean diet adherence and metabolic outcomes were examined using correlation analyses and multivariable regression models adjusted for relevant confounders. **Results**: Most participants showed low adherence to the Mediterranean diet. A significant inverse association was observed between Mediterranean diet adherence and hemoglobin A1c (HbA1c) levels, with individuals scoring ≤35 on the MDS demonstrating higher HbA1c levels. Similar trends were observed in the lowest tertile of adherence. Notably, each one-point increase in MDS predicted a 0.13% reduction in HbA1c. In multivariable regression analyses, Mediterranean diet adherence remained the strongest predictor of glycemic control, independent of age, body mass index (BMI), sex, smoking status, physical activity and the number of antidiabetic treatments. Higher adherence was also significantly associated with lower low-density lipoprotein cholesterol (LDL-C) and triglyceride (TG) levels, as well as higher high-density lipoprotein cholesterol (HDL) concentrations. **Conclusions**: Greater adherence to the Mediterranean diet is independently associated with improved glycemic regulation and a more favorable lipid profile in adults with T2D. These findings support the Mediterranean diet as a valuable non-pharmacologic strategy for optimizing metabolic outcomes in people with T2D.

## 1. Introduction

Diabetes is a chronic metabolic disorder characterized by persistent hyperglycemia resulting from defects in insulin secretion, insulin action, or both. Its prevalence has reached epidemic proportions, affecting hundreds of millions of people worldwide and imposing a significant burden on public health systems [1]. In 2021, it was estimated that 537 million adults (10.5% of the population) were living with diabetes globally. This number is expected to increase to 643 million (11.3%) by 2030 and to 783 million (12.2%) by 2045 [2]. Among its forms, type 2 diabetes (T2D) is the most common and is closely linked to lifestyle factors such as diet, physical inactivity, and obesity [3]. According to the International Diabetes Federation (IDF), the prevalence of T2D in Greece was estimated at approximately 9.6% of the adult population in 2021, highlighting a substantial public health challenge [2].

While pharmacological therapies remain central to diabetes management, over the past decades, non-pharmacological interventions, particularly dietary strategies, have been increasingly recognized as pivotal for long-term metabolic control [4]. Dietary interventions have garnered growing attention as modifiable risk factors for the onset and progression of T2D [5]. Various dietary patterns, such as low-carbohydrate, low–glycemic index, vegetarian, and Dietary Approaches to Stop Hypertension (DASH) diets, have been extensively studied for their potential to improve glycemic control and reduce diabetes-related complications [6,7,8]. However, the traditional Mediterranean diet, characterized by high consumption of fruits, vegetables, legumes, whole grains, and olive oil, along with moderate intake of fish and poultry, has been established as the dietary pattern with the most substantial and well-documented benefits, particularly in long-term glucose regulation, enhanced insulin sensitivity, and the reduction in cardiovascular risk [9,10].

Evidence indicates that adopting a Mediterranean diet is associated with improvements in glycemic control, reflected by reductions in hemoglobin A1c (HbA1c) levels [11,12]. A meta-analysis by Schwingshackl et al. reported a mean HbA1c reduction of 0.32% [11], while Esposito et al. demonstrated decreases of up to 0.6%, along with reductions in fasting plasma glucose (FPG) ranging from 7 to 40 mg/dL [12]. Furthermore, findings from the ATTICA cohort showed that higher adherence to the Mediterranean diet was linked to a 21% lower 20-year risk of developing T2D. The Mediterranean diet is also associated with long-term improvements in lipidemic profile, including lower levels of total cholesterol (TC), low-density lipoprotein cholesterol (LDL-C), lower triglycerides (TG) and lower non-high-density lipoprotein cholesterol (non-HDL-C) at baseline and at 10-year follow-up [13]. A healthy lipidemic profile is a strong protective factor against cardiovascular disease. Nevertheless, adherence to the Mediterranean diet among people with T2D varies considerably and is generally moderate to low, while its impact on glycemic control in real-world settings remains under investigation [14].

Several tools have been developed to assess dietary adherence [15], among which the total Mediterranean Diet Score (MDS) by Panagiotakos et al. has some of the strongest supporting evidence [16]. The MDS evaluates 11 key components of the Mediterranean diet, including non-refined cereals, fruits, vegetables, olive oil, fish, full-fat dairy products, and alcohol consumption. MDS ranges from 0 to 55, with higher scores indicating greater adherence [16]. This scoring system was applied in the present study to assess compliance with Mediterranean diet guidelines.

The aim of the present study was to evaluate adherence to the Mediterranean diet among people with T2D attending the Diabetes Center of Laiko University Hospital (Athens, Greece) and to examine its association with glycemic control, as reflected by HbA1c levels, as well as its effects on participants’ lipidemic profiles.

## 2. Materials and Methods

### 2.1. Study Design and Participants

This cross-sectional study was conducted at the outpatient clinics of Laiko General Hospital, Athens, Greece, from May 2024 to September 2024. A total of 100 adults with a confirmed diagnosis of T2D who attended regular follow-ups were consecutively recruited. Inclusion criteria were a diagnosis of T2D for at least 6 months, age 18–85 years and regular visits to the outpatient clinic of the hospital. Exclusion criteria were age above 85 years, recent acute illnesses and patients who did not visit the outpatient clinic regularly.

### 2.2. Ethical Approval

The study was conducted in accordance with the Declaration of Helsinki (2000). Written informed consent was obtained from all participants prior to enrollment. The study protocol was approved by the Ethics Committee of Laiko General Hospital (Approval Number: 36/12-02-2024).

### 2.3. Data Collection

#### 2.3.1. Sociodemographic and Lifestyle Characteristics

Participants reported their age, sex, and educational level (primary, secondary, tertiary). Lifestyle information included smoking status (current vs. non-smoker; non-smokers were defined as people who had not smoked for at least one month prior to the interview) and physical activity. Physical activity was assessed using metabolic equivalents of task (MET-min/week) and categorized as low (<600 MET-min/week), moderate (≥600 MET-min/week), or high (≥1500 MET-min/week or ≥3000 MET-min/week for combined activities).

#### 2.3.2. Anthropometric Measurements

Body weight was measured to the nearest 0.1 kg with participants lightly clothed and after a minimum 3 h fast, and height to the nearest 0.1 cm using a stadiometer. Body mass index (BMI) was calculated as kg/m^2^, with overweight defined as 25–29.9 kg/m^2^ and obesity as ≥30 kg/m^2^. Waist circumference (WC) was measured at the narrowest part, 1 cm above the umbilicus, and hip circumference (HC) at the widest part. Waist-to-hip ratio (WHR) was calculated from these measurements.

#### 2.3.3. Clinical and Biochemical Parameters

Blood pressure (BP) was measured after 5 min of rest in a seated position using an automated sphygmomanometer. Laboratory assessments included FPG, HbA1c, lipid profile with TC, LDL-C, HDL-C, and TGs, urea, creatinine, aspartate aminotransferase (AST), alanine aminotransferase (ALT), and electrolytes, including sodium (Na^+^) and potassium (K^+^). Estimated glomerular filtration rate (eGFR) was calculated using the 2021 CKD-EPI Creatinine equation.

#### 2.3.4. Dietary Assessment

Adherence to the Mediterranean diet was evaluated using the validated MDS. The questionnaire assessed the frequency of consumption of 11 food groups typical of the Mediterranean diet over the past month. The total score ranged from 0 (minimal adherence) to 55 (maximal adherence), with higher scores indicating greater adherence. For foods considered characteristic of the Mediterranean diet—namely non-refined cereals (whole-grain bread, pasta, rice; ~1 slice bread or ^1^/_2_ cup cooked grains per serving), fruits (1 medium fruit or ~150–200 g), vegetables (1 cup raw or ^1^/_2_ cup cooked; ~100–150 g), legumes (1 cup cooked; ~150–200 g), potatoes (1 medium potato; ~150 g), fish (100–150 g cooked), and olive oil (evaluated qualitatively as the main culinary fat)—the score increases with higher consumption frequency, from 0 for never/rare consumption to 5 for daily consumption (or habitual exclusive use, in the case of olive oil). For foods not typical of the Mediterranean diet, including red meat and meat products (100–120 g cooked red meat or 50–60 g processed meat per serving), poultry (100–120 g cooked), and full-fat dairy products (1 cup whole milk or yogurt or 30–40 g cheese), the scoring is reversed, such that lower consumption frequencies receive higher scores (5 for never/rare intake and 0 for daily intake). Alcohol consumption, traditionally reflecting wine intake, is scored separately using 100 mL wine equivalents per day, with moderate consumption (100–300 mL/day, approximately 1–3 glasses) receiving the maximum score of 5, while both abstinence and excessive intake receive lower scores. Consumption frequencies are categorized as never, rare, ≤1 time/week, 2–3 times/week, 4–6 times/week, or daily, corresponding to scores from 0 to 5 depending on whether the food is considered beneficial or non-beneficial within the Mediterranean pattern.

### 2.4. Statistical Analysis

Continuous variables are presented as mean ± standard deviation (SD), and categorical variables as counts and percentages. The study population was divided into two groups according to the median MDS. Between-group comparisons were performed using independent-sample *t*-tests or Kruskal–Wallis tests, as appropriate. Spearman’s correlation and linear regression analyses were conducted with MDS as a continuous variable. Multiple linear regression models were applied to adjust for potential confounders, including age, BMI, and smoking status. Standardized β coefficients and 95% confidence intervals (CI) are reported. A *p*-value < 0.05 was considered statistically significant. Analyses were performed using the statistical package for the social sciences (SPSS; IBM Corp. IBM SPSS Statistics for Windows. Version 29.0. Armonk, NY, USA). Figures were created with RStudio (R version 4.3.1).

### 2.5. Data Availability

Individual-level data are not publicly available due to institutional restrictions. Aggregated data may be requested from the corresponding author upon reasonable request.

### 2.6. Use of Generative Artificial Intelligence

No generative artificial intelligence (GenAI) tools were used in study design, data collection, analysis, or interpretation.

## 3. Results

### 3.1. Sample Characteristics

A total of 100 patients with T2D were included in the study. The sample was balanced in terms of gender, comprising 54 men and 46 women. The mean age was 62 ± 10 years, with the youngest participant aged 41 years and the oldest 81 years. Regarding lifestyle characteristics, physical activity levels were predominantly low. Specifically, 68% of patients reported light physical activity, 21% moderate activity, and only 11% high levels of activity. About smoking, 78% were classified as non-smokers and 22% as active smokers at the time of assessment.

Anthropometric measurements indicated a high prevalence of obesity. The mean body weight was 92.2 ± 10.1 kg, corresponding to a mean BMI of 31.7 ± 7.0 kg/m^2^. More than two-thirds of the population had a BMI > 30 kg/m^2^, classifying them as patients with obesity, while the remainder were predominantly overweight. The mean WHR was 0.96 ± 0.12, consistent with increased cardiometabolic risk. Biochemical analysis revealed a mean HbA1c of 6.7 ± 1.1% and a mean FPG of 115 ± 20 mg/dL. Lipid measurements indicated a mean TC of 173 ± 28 mg/dL, LDL-C of 93 ± 23 mg/dL, HDL-C of 46 ± 10 mg/dL, and TGs of 127 ± 37 mg/dL.

Medication use was extensive, consistent with the comorbidities often observed in people with diabetes. Forty-three percent of the total sample (43%) was on antihypertensive therapy, including β-blockers (25%), angiotensin-converting enzyme inhibitors (ACEIs) or angiotensin receptor blockers (ARBs) (12%), calcium-channel blockers (CCBs) (4%), and α-blockers (2%). Lipid-lowering therapy was reported by 62% of patients, mainly in the form of statins. All patients received pharmacologic antidiabetic treatment, with multiple treatment combinations used, as is typical in T2D management.

### 3.2. Adherence to the Mediterranean Diet

The mean MDS across the sample was 34 ± 4 points. Using the sample median (35 points) as a threshold, 54% of participants were classified as having low adherence (≤35 points), while 46% achieved high adherence (>35 points). Because several participants shared identical MDS values, when categorized into tertiles, there were 31 individuals in the low-adherence tertile (≤32 points), 40 in the medium-adherence tertile (MDS 33–37), and 29 in the high-adherence tertile (>38 points).

The characteristics of participants stratified by high and low adherence to the Mediterranean diet are summarized in Table 1.

### 3.3. Association Between Mediterranean Diet Adherence and Glycemic Control

When the MDS was analyzed as a binary variable, patients in the high-adherence group exhibited significantly lower HbA1c compared to those in the low-adherence group (6.1 ± 0.6% vs. 7.2 ± 1.1%; *p* = 0.028). Similarly, FPG was lower among high-adherence participants (110 ± 22 mg/dL vs. 121 ± 17 mg/dL; *p* = 0.047).

**Figure 1:**
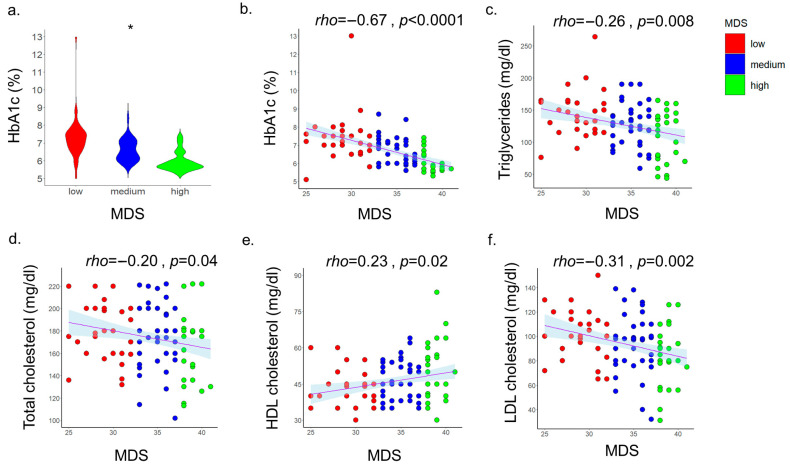
Violin plot showing the distribution of HbA1c across tertiles of MDS. Because several participants shared identical MDS values, the final groups included 31 individuals in the low-adherence tertile, 40 in the medium-adherence tertile, and 29 in the high-adherence tertile (**a**). Correlations between MDS and HbA1c, TG, total cholesterol, HDL cholesterol and LDL cholesterol values (**b**–**f**). Abbreviations: HbA1c: hemoglobin A1c; HDL: high-density lipoprotein; LDL: low-density lipoprotein; MDS: Mediterranean Diet Score. * denotes significant difference in HbA1c levels among the 3 groups.

Analysis by tertiles revealed a clear gradient in glycemic control: HbA1c was highest in the lowest adherence tertile (mean 7.2%), intermediate in the middle tertile (6.6%), and lowest in the highest tertile (6.1%) (*p* for trend <0.05, Kruskal–Wallis test) (Figure 1a). In line with this, in univariate analysis, there was a significant inverse association between MDS and HbA1c (Spearman’s ρ = −0.67, *p* < 0.001) (Figure 1b). Linear regression analysis indicated that each one-point increase in MDS predicted a 0.13% reduction in HbA1c (β = −0.13, *p* < 0.001). Multiple linear regression analyses were performed to control for potential confounders, including age, sex, BMI, smoking status, physical activity and the number of antidiabetic treatments. In these adjusted models, MDS was the only significant predictor of HbA1c [standardized coefficient (st.) β = −0.51, *p* < 0.001]. Table 2 displays in detail the antidiabetic and lipid-lowering treatments used in the two groups. Although there were no significant differences in the types of drugs used between the two groups, there were more patients in the low MDS group who used insulin treatment or more than 3 or more than 4 antidiabetic drugs compared to the high MDS group, although these findings were not significant.

We performed sensitivity analyses excluding patients who received more advanced pharmacotherapy to treat diabetes. When excluding patients receiving insulin, in the remaining 66 patients, the inverse association between HbA1c and MDS remained significant and was even stronger (Spearman’s ρ = −0.64, *p* < 0.001). Likewise, when excluding patients who needed more than 3 drugs to treat their diabetes, there was a strong inverse association between HbA1c and MDS (N = 83, Spearman’s ρ = −0.67, *p* < 0.001).

### 3.4. Association Between Mediterranean Diet Adherence and Lipid Profile

In addition to effects on glycemic control, Mediterranean diet adherence was associated with favorable lipid parameters. In univariate analysis, there were significant associations between MDS and TG, TC, LDL-C and HDL-C (Figure 1c–f).

In line with this finding, when the study population was stratified by adherence to the Mediterranean diet, participants in the high-adherence group showed lower TC (167 ± 30 mg/dL vs. 180 ± 26 mg/dL, *p* = 0.04), LDL-C (86 ± 25 mg/dL vs. 99 ± 20 mg/dL, *p* = 0.008), TG levels (113 ± 38 mg/dL vs. 138 ± 34 mg/dL, *p* = 0.004) and higher HDL-C levels (49 ± 11 mg/dL vs. 44 ± 8 mg/dL, *p* = 0.049) compared to the low-adherence group. When accounting for treatment with hypolipidemic medications, the high-adherence group still exhibitedlower TC values (st. β = −0.25, *p* = 0.01), lower LDL-C values (st. β = −0.33, *p* = 0.001), lower TG (st. β = −0.27, *p* = 0.005), and higher HDL-C levels (st. β = 0.22, *p* = 0.03), compared to the low-adherence group.

Since 4 participants in the high-adherence group received the most potent combination of lipid-lowering therapy (high-intensity statin plus ezetimibe), whereas none in the low-adherence group did (Table 2), we conducted a sensitivity analysis excluding these 4 individuals. Even after their exclusion, participants in the high-adherence group continued to show higher HDL-C levels (49 ± 11 vs. 44 ± 8, *p* = 0.049), lower LDL-C levels (88 ± 22 vs. 99 ± 20, *p* = 0.01), and lower TGs (110 ± 38 vs. 138 ± 34, *p* = 0.001), with no significant difference in TC (*p* = 0.06).

### 3.5. Νο Association Between Mediterranean Diet Adherence and BMI, Blood Pressure, Renal Function, or Aminotransferases

We could not detect any difference in BMI, systolic or diastolic BP, eGFR, AST, or ALT levels between the high-adherence and the low-adherence MDS groups. Likewise, there was no association between MDS and these parameters.

## 4. Discussion

This observational study of Greek adults with T2D demonstrated that higher adherence to the traditional Mediterranean diet is a strong and independent predictor of improved glycemic and lipidemic control. These findings align with previous evidence indicating that the Mediterranean dietary pattern is one of the most effective nutritional approaches for mitigating cardiometabolic risk [10]. A central result of the present study is the significant inverse association between MDS and HbA1c, where each one-point increase in MDS corresponded to an approximate 0.13% reduction in HbA1c after adjustment for age, sex, BMI, smoking status, physical activity, and the number of glucose-lowering medications. In these adjusted models, MDS remained the strongest independent predictor of HbA1c (st. β = −0.49, *p* < 0.0001), while none of the other variables demonstrated significant independent associations.

Even moderate improvements in diet adherence may translate into clinically meaningful differences in glycemic control. This result is consistent with earlier epidemiological research showing that Mediterranean dietary interventions reduce HbA1c, FPG, and insulin resistance in people with diabetes [17]. Comparable benefits have been demonstrated in major randomized trials, such as the PREDIMED study, where adherence to a Mediterranean diet enriched with olive oil or nuts resulted in a diabetes incidence of approximately 10%, whereas the corresponding rate in the placebo group reached about 18% after a median follow-up of four years [18]. Similar improvements have also been observed in people with prediabetes, with additional gains derived from structured exercise programs in reducing the risk of developing T2D [19,20].

Beyond glycemic regulation, high Mediterranean diet adherence was associated with lower TGs, LDL-C levels and TC levels, and higher HDL-C levels. When accounting for treatment with lipid-lowering medications, high Mediterranean diet adherence was still associated with a better profile of all parameters (TGs, LDL-C, HDL-C, TC). However, it should be noted that in the study population, LDL-C levels were frequently above target, which is defined as <70 mg/dL for patients at high cardiovascular risk and <55 mg/dL for those at very high cardiovascular risk [21]. These findings are consistent with previous studies in Greek populations, including the ATTICA and the EPIC cohort study, which identified more favorable lipid profiles among people with higher adherence to the Mediterranean diet [13,22].

Improvements in LDL-C are particularly significant, given the well-established association between LDL-C reduction and cardiovascular risk mitigation, including in primary prevention settings [23,24,25]. In contrast, the clinical relevance of changes in HDL-C and TGs remains unclear, particularly because pharmacologic interventions that increase HDL-C or decrease TGs, such as cholesteryl ester transfer protein (CETP) inhibitors and pemafibrate, respectively, failed to improve cardiovascular outcomes [26]. An important exception is the Reduction of Cardiovascular Events with Icosapent Ethyl–Intervention Trial (REDUCE-IT), which established purified EPA (icosapent ethyl) as an effective therapy for hypertriglyceridemia, demonstrating significant reductions in ischemic events, including cardiovascular death, compared with placebo [27]. Evidence also supports that HbA1c changes associated with Mediterranean diet adherence may explain up to 20% of lipid-related benefits [28]. The cross-sectional nature of the present study did not permit evaluation of whether the favorable lipid changes observed among people with high adherence reflect broader metabolic restructuring associated with improved diet quality or represent specific lipid alterations that directly contribute to cardioprotection.

The nutrient composition of the Mediterranean diet provides a mechanistically plausible explanation for the metabolic improvements observed. High intake of monounsaturated and polyunsaturated fats derived from olive oil and nuts, combined with the anti-inflammatory and antioxidant properties of plant-based foods, supports insulin sensitivity and promotes glucose homeostasis [29]. Also, the Mediterranean diet is known for its phenolic compounds, which contribute to improved glycemic control and a reduced risk of T2D [30]. Proposed mechanisms include antioxidant and anti-inflammatory effects, enhancement of insulin signaling pathways and modulation of gut microbiota composition. These mechanisms collectively improve insulin sensitivity and reduce postprandial glucose excursions. Polyphenols from fruits and vegetables, including anthocyanins, quercetin and catechins, have also been associated with improved glycemic markers. Clinical trials and observational studies indicate that higher intake of these compounds is linked to lower FPG and improved insulin sensitivity. Resveratrol, mainly found in grapes and red wine, has shown antihyperglycemic effects in some randomized controlled trials, although findings remain inconsistent across studies, likely due to differences in dosage, duration and study populations [31].

These nutritional characteristics also contribute to improvements in lipid metabolism, reinforcing the concept that diet quality exerts meaningful effects on metabolic outcomes independently of caloric intake [32]. Conversely, dietary patterns rich in saturated and trans fats have been shown to increase insulin resistance and accelerate the progression of T2D [33]. What is more, the Mediterranean diet is rich in fiber, which helps regulate blood sugar levels. Fiber can help delay gastric emptying, which slows down glucose absorption and, as a result, leads to lower serum insulin concentrations. Through its high content of fiber, this diet can lead to satiety, so it helps in an indirect way to maintain a normal body weight or even to lose weight, which helps to regulate blood sugar levels [34]. What is interesting is the nutrigenetic interaction between carbohydrates and candidate genes in T2D. For example, carriers of risk alleles of the *TCF7L2* gene tend to have impaired insulin secretion and higher postprandial glucose levels, particularly when consuming diets high in refined carbohydrates or with a high glycemic index. In contrast, whole-grain and low-glycemic-load carbohydrate sources may mitigate this genetic risk. Evidence suggests that both carbohydrate quality and quantity interact with genetic background to modulate T2D risk and glycemic outcomes [35].

Despite the beneficial associations identified for glycemic and lipid parameters, no significant associations were detected between Mediterranean diet adherence and either BP or BMI. The limited sample size and cross-sectional design may have restricted the ability to detect more subtle effects. It is plausible that, in people with established diabetes, the metabolic influence of diet manifests more readily in glycemic and lipid indices than in anthropometric or hemodynamic variables, which may require sustained behavioral change over longer periods to reveal measurable alterations.

A particularly notable finding of the present investigation is the overall low adherence to the Mediterranean diet among this urban Greek cohort. Less than one-third of participants demonstrated high adherence to the Mediterranean diet. This observation aligns with accumulating evidence indicating a steady decline in traditional dietary habits across both Mediterranean and non-Mediterranean countries, driven by modernization, urbanization, increased access to ultra-processed foods, and shifting lifestyle patterns [33,36]. This trend is especially concerning for people with T2D, a population in which dietary patterns exert a substantial influence on disease progression and cardiovascular risk [37]. It is also noteworthy that those using a greater number of antidiabetic medications tended to show numerically lower adherence, although this association was not statistically significant. One possible explanation for the present trend is that newer antidiabetic agents, particularly glucagon-like peptide-1 receptor agonists (GLP-1 RAs) and secondary sodium–glucose co-transporter 2 inhibitors (SGLT2-i), can induce clinically meaningful weight loss and glycemic improvements, potentially fostering a sense of complacency regarding adherence to dietary recommendations [38]. However, this hypothesis requires exploration through larger, longitudinal studies.

The present study has several limitations that merit consideration. The cross-sectional design precludes causal inference, and the possibility of reverse causality cannot be excluded. Dietary assessment relied on self-reported measures, which are susceptible to recall bias and social desirability bias. The relatively small sample size may have limited the ability to detect more modest associations, particularly those involving BMI, BP, or subgroup analyses. Furthermore, recruitment from a single tertiary center in Athens may restrict generalizability to other regions of Greece with distinct cultural or socioeconomic characteristics. Additionally, lifestyle factors such as detailed physical activity, stress, and socioeconomic status were not comprehensively assessed despite their known influence on dietary patterns and metabolic health.

On the other hand, this study has several important strengths. It reflects a real-world clinical population, which enhances external validity and provides valuable insight into the typical dietary behaviors of Greek adults with T2D living in urban areas. Mediterranean diet adherence was assessed using a validated score [16]. Furthermore, multivariable regression adjusting for major confounders, including age, BMI, smoking, METs, and medication use, provides robust evidence supporting the independent association between Mediterranean diet adherence and improved metabolic outcomes [39,40].

Collectively, the present findings highlight the central role of dietary quality in the management of T2D, despite significant advances in pharmacotherapy. People with diabetes frequently encounter barriers to adopting and maintaining healthy dietary patterns, including limited nutritional literacy, financial constraints, insufficient meal-planning skills, and inadequate access to dietary counseling [41]. These challenges emphasize the need for personalized dietary education and long-term behavioral support within clinical practice. Future research should employ longitudinal designs or randomized controlled trials to clarify causal pathways connecting Mediterranean diet adherence with glycemic and lipid outcomes. The incorporation of dietary biomarkers, digital tools for monitoring dietary intake, and objective adherence measures could enhance accuracy in diet assessment. Expanding recruitment to diverse geographic regions would also facilitate the identification of cultural and socioeconomic determinants of dietary behaviors. Ultimately, integrating structured dietary interventions with broader lifestyle modifications, including increased physical activity, has the potential to yield synergistic benefits and establish the Mediterranean diet as a cost-effective and sustainable cornerstone in the prevention and management of T2D.

## 5. Conclusions

This observational real-world study reinforces previous evidence supporting the beneficial impact of the Mediterranean dietary pattern on glycemic and lipidemic regulation in adults with T2D. The observed inverse association between adherence to the Mediterranean diet and HbA1c levels highlights the critical role of dietary habits in diabetes management, even in populations already under pharmacologic treatment. These results underline the necessity of integrating structured dietary education and lifestyle interventions into routine diabetes care, aiming to enhance long-term metabolic outcomes and quality of life. Promoting adherence to the Mediterranean diet from an early stage of disease progression could serve as a valuable, non-pharmacologic strategy for improving glycemic control and reducing diabetes-related complications.

## Figures and Tables

**Table 1:** Baseline demographic, anthropometric and biochemical characteristics of the study population (N = 100).

	Low MDS Group N = 54	High MDS Group N = 46	*p* Value
Age (years)	61 ± 11	64 ± 9	0.08
Weight (kg)	93.5 ± 23.8	90.6 ± 25.1	0.5
Physical activity (METs-min/week)	642 ± 834	575 ± 784	0.6
BMI (kg/m^2^)	31.9 ± 7.1	31.5 ± 7.0	0.8
HbA1c (%)	7.2 ± 1.1	6.1 ± 0.6	<0.0001
FPG (mg/dL)	121 ± 17	110 ± 22	0.001
Urea (mg/dL)	38 ± 10	39 ± 11	0.7
Creatinine (mg/dL)	0.8 ± 0.2	0.8 ± 0.3	0.8
eGFR (mL/min/1.73 m^2^)	97 ± 18	91 ± 18	0.6
TC (mg/dL)	180 ± 26	167 ± 30	0.04
Triglycerides (mg/dL)	138 ± 34	113 ± 38	0.004
HDL-C (mg/dL)	44 ± 8	49 ± 11	0.049
LDL-C (mg/dL)	99 ± 20	86 ± 25	0.008
AST (U/L)	23 ± 10	21 ± 9	0.5
ALT (U/L)	23 ± 10	21 ± 7	0.4
Na^+^ (mEq/L)	138 ± 4	138 ± 4	>0.9
K^+^ (mEq/L)	4.0 ± 0.3	4.0 ± 0.3	0.4
SBP (mmHg)	132 ± 10	131 ± 10	0.6
DBP (mmHg)	84 ± 6	84 ± 9	>0.9
W/H ratio	0.96 ± 0.12	0.96 ± 0.13	0.9

Abbreviations: ALT: alanine aminotransferase; AST: aspartate aminotransferase; BMI: body mass index; DBP: diastolic blood pressure; eGFR: estimated glomerular filtration rate; FPG: fasting plasma glucose; HbA1c: glycated hemoglobin; HDL-C: high-density lipoprotein cholesterol; LDL-C: low-density lipoprotein cholesterol; MDS: Mediterranean Diet Score; METs: metabolic equivalents of task; SBP: systolic blood pressure; TC: total cholesterol; W/H ratio: waist-to-hip ratio.

**Table 2:** Description of antidiabetic and lipid-lowering treatments of the study population (N = 100) in low vs. high MDS groups.

	Low MDS Group N = 54	High MDS Group N = 46	*p* Value
Metformin only (N)	11	11	0.7
Metformin and SGLT2-i (N)	8	4	0.3
Metformin and DPP4-i (N)	2	3	0.5
Metformin and GLP-1 RA (N)	4	1	0.2
Metformin and basal-bolus (N)	1	0	0.4
Metformin, DPP4-i and SGLT2-i (N)	3	3	0.8
DPP4-i (N)	1	2	0.5
Basal insulin only (N)	1	0	0.4
Basal insulin and metformin (N)	0	1	0.3
Basal insulin and DPP4-i (N)	2	1	0.7
Basal insulin and GLP-1 RA (N)	1	3	0.2
Basal insulin, metformin, SGLT2-i (N)	2	2	0.9
Basal insulin, GLP-1 RA, SGLT2-i (N)	1	2	0.5
Basal insulin, metformin, DPP4-i (N)	1	0	0.4
Metformin, SGLT2-i, GLP-1 RA (N)	4	5	0.5
Basal insulin, metformin, SGLT2-i, GLP-1 RA (N)	6	4	0.7
Basal insulin, metformin, GLP-1 RA, TZD (N)	1	0	0.4
Basal-bolus insulin, metformin, SGLT2-i, GLP-1 RA (N)	2	2	0.9
SGLT2-i and GLP-1 RA (N)	1	1	0.9
SU, metformin, DPP4-i, SGLT2-i (N)	2	1	0.6
≥3 drugs (N)	24	22	0.74
≥4 drugs (N)	10	7	0.66
Insulin treatment (N)	18	16	0.88
Lipid-lowering treatment			
Treatment with low-intensity statin only (N)	8	7	0.96
Treatment with high-intensity statin only (N)	13	18	0.10
Treatment with low-intensity statin and ezetimibe (N)	7	5	0.75
Treatment with high-intensity statin and ezetimibe (N)	0	4	0.03
Treatment with ezetimibe only (N)	0	0	-

Abbreviations: DPP4-i: dipeptidyl peptidase IV inhibitors; GLP-1 RA: glucagon-like peptide-1 receptor agonist; MDS: Mediterranean Diet Score; SGLT2-i: sodium glucose co-transporter 2 inhibitor; SU: sulfonylureas; TZD: thiazolidinedione. N: stands for the number of subjects receiving the specific medication. High-intensity statin: atorvastatin 40 mg or higher, or rosuvastatin 20 mg or higher.

## Data Availability

All data generated or analyzed during this study are included in the article. Further inquiries can be directed to the corresponding author.

## Abbreviations

95% CI: 95% confidence interval; ACEI: angiotensin-converting enzyme inhibitor; ALT: alanine aminotransferase; ARB: angiotensin receptor blocker; AST: aspartate aminotransferase; BMI: body mass index; BP: blood pressure; CCB: calcium-channel blocker; CETP: cholesteryl ester transfer protein; DASH: Dietary Approaches to Stop Hypertension; DPP4-i: dipeptidyl peptidase-4 inhibitor; eGFR: estimated glomerular filtration rate; FPG: fasting plasma glucose; GenAI: generative artificial intelligence: GLP-1 RA: glucagon-like peptide-1 receptor agonist; HbA1c: hemoglobin A1c; HC: hip circumference; HDL-C: high-density lipoprotein cholesterol; IDF: International Diabetes Federation; LDL-C: low-density lipoprotein cholesterol; MDS: Mediterranean Diet Score; MET: metabolic equivalent of task; REDUCE-IT: Reduction of Cardiovascular Events with Icosapent Ethyl–Intervention Trial; SD: standard deviation; SGLT2-i: sodium–glucose co-transporter-2 inhibitor; SU: sulfonylurea; T2D: type 2 diabetes; TC: total cholesterol; TG: triglyceride; TZD: thiazolidinedione; WC: waist circumference; WHR: waist-to-hip ratio.
